# Bilateral Humeral Head Avascular Necrosis: A Rare Presentation in Sickle Cell Trait

**DOI:** 10.7759/cureus.44006

**Published:** 2023-08-23

**Authors:** Shashank Banait, Tanvi Banait, Shivani Shukla, Supriya Mane, Jyoti Jain

**Affiliations:** 1 Department of Ophthalmology, Jawaharlal Nehru Medical College, Datta Meghe Institute of Higher Education & Research (DMIHER), Wardha, IND; 2 Department of Medicine, Jawaharlal Nehru Medical College, Datta Meghe Institute of Higher Education & Research (DMIHER), Wardha, IND; 3 Department of Medicine, Mahatma Gandhi Institute of Medical Sciences, Wardha, IND

**Keywords:** hemoglobinopathy, sickle cell trait., sickle cell disease, humeral head osteonecrosis, avascular necrosis

## Abstract

Sickle cell disease (SCD) affects millions of people worldwide. It is an autosomal recessive hemoglobinopathy that occurs due to a point mutation in the sixth codon that replaces glutamic acid with valine in the beta-globin chain. Avascular necrosis (AVN), also known as osteonecrosis, is one of its complications. In this report, we present a case of a 25-year-old female with sickle cell trait without any comorbidities who presented to us with pain in both shoulder joints for three months and was diagnosed with AVN in bilateral shoulder joints. Appropriate treatment can dramatically reduce pain and improve the quality of life for these patients. This case drew our attention due to its rare presentation.

## Introduction

Millions of people worldwide suffer from sickle cell disease (SCD), and India has the second-highest proportion of SCD patients after sub-Saharan Africa [1]. While patients with sickle cell trait only inherit one allele of the hemoglobin beta gene (heterozygous), patients with sickle cell anemia are homozygous for two copies of the faulty allele. Patients with SCD experience a range of symptoms, from mild weakness and muscle stiffness to extreme pain, fever, rheumatism, and chronic organ failure; however, patients with sickle cell trait do not have such severe symptoms. Avascular necrosis (AVN) refers to the death of the osteocytes of the bone caused by ischemia and thereby leading to the failure of the essential bone marrow. The sites most susceptible to AVN are the femoral head, the femoral condyles, the head of the humerus, the capitulum, and the adjoining parts of the scaphoid and talus [2]. Radiographically, AVN on both shoulders is found in approximately one-third of SCD patients. However, hip and shoulder AVN in sickle cell trait is a rare entity [3]. According to the disease pathophysiology, it occurs because glutamic acid (a hydrophilic amino acid) is substituted by valine (a hydrophobic amino acid). This mutation results in the protein structure producing an external hydrophobic end. Red blood corpuscles (RBCs) "sickle" after vicious cycles of polymerization and depolymerization, which cause the RBC membrane to harden. These twisted, rigid RBCs obstruct small blood vessels in multiple organs, leading to the various clinical manifestations and crises associated with SCD [4]. The management of AVN of the shoulder joint due to SCD is difficult due to the natural course of the disease, and progression of osteonecrosis, and the collapse of the humeral head occurs despite early surgical interventions, i.e., core decompression [5].

## Case presentation

**Figure 1:**
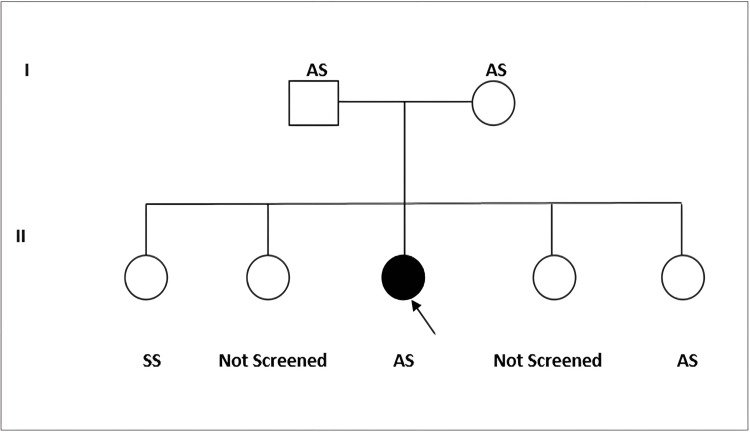
Pedigree chart showing positive family history in the index patient with one of the siblings having sickle cell anemia and another sibling having sickle cell trait SS: sickle cell disease; AS: sickle cell trait

A 25-year-old woman without any prior history of diabetes or any autoimmune disease presented to our hospital with the main complaint of pain in both shoulder joints for three months. She reported that work and raising the joint aggravated her shoulder pain, and analgesic drugs did not help alleviate the pain. She did not experience discomfort or morning stiffness in any other minor or big joints. There was no prior history of fever, falls, joint trauma, steroid use, alcohol abuse, anti-resorptive or bisphosphonate medication use, radiation therapy, or similar complaints of that nature. As for her family history, both her parents had sickle cell trait (AS pattern), and among her siblings, one sister had SCD (SS pattern), another had sickle cell trait (AS pattern), and the other two sisters had not yet been screened for the disease*. *The patient was diagnosed to have sickle cell trait (AS pattern) based on high-performance liquid chromatography (HPLC) during this admission* *(Figure 1).

The patient was lean and had mild pallor and bilateral pitting pedal edema on general examination. Icterus, cyanosis, lymphadenopathy, or elevated jugular venous pressure (JVP) were not observed. When her shoulder joints were examined locally, both joint lines were painful, with the left joint being more so. Both shoulder joints were painful to move in any way. On the right and left sides, there was flexion of 100° and 80°, abduction of 120° and 50°, and extension of 5-10° and 3°, respectively. Due to considerable pain on the left side, neither internal nor external rotation could be performed; however, on the right side, 10° of internal and 40-50° of external rotation were seen.

Her systemic examination did not reveal any abnormalities. Her hemogram revealed hemoglobin levels of 7.1 g/dl, a total leukocyte count of 15,200/cu mm, platelet counts of 5.77 lakhs/UL, and a peripheral smear showed anisopoikilocytosis, normocytic to microcytic, hypochromic RBCs, sickled red cell presence, macrocytes, target cells, and polychromatic cells, along with a drop in total RBC count (2.56 million/mm^3^). Her blood sugar, electrolytes, liver function, and chest X-ray were all normal. She tested negative for serological tests for antinuclear antibodies (ANA), anti-double-stranded DNA (Anti-dsDNA), antibodies to human immunodeficiency virus (HIV), rheumatoid arthritis (RA) factor, and anti-cyclic citrullinated peptide antibody (Anti-CCP). Table1shows the laboratory values during her hospital course.

**Table 1:** Patient’s laboratory values on admission

Laboratory tests	Values on admission	Reference values
Complete blood count		
Hemoglobin	7.1	11-14 gm/dL
Mean corpuscular volume (MCV)	72	80-100 femtolitre
Mean corpuscular hemoglobin (MCH)	24.8	27-31 picogram
Mean corpuscular hemoglobin concentration (MCHC)	34.2	33-37 gm/dl
Red cell distribution width (RDW)	25.4	11.6-13.7%
White blood cells	15.2	4-11 (in K/uL)
Neutrophils	81.8	60-70%
Lymphocytes	13.6	20-30%
Platelets	577	150-350 (in lakhs/uL)
Other hematological tests		
Reticulocyte count	1.8	0.5-3%
Serum lactate dehydrogenase (LDH)	288	230-460 (U/L)
Erythrocyte sedimentation rate (ESR)	20 mm/hour	<20 mm/hour
Glucose-6-phosphate dehydrogenase (G6PD)	Non-deficient	Non-deficient
Renal function tests		
Serum creatinine	0.95	0.72-1.18 mg/dl
Serum urea	23	17-43 mg/dl
Serum sodium	137	136-146 mEq/L
Serum potassium	4.35	3.5-5.1 mEq/L
Serum uric acid	5.70	3.5-7.2 mg/dl
Liver function tests		
Serum protein	7.76	6.6-8.3 gm/dl
Serum albumin	3.29	3.5-5.2 gm/dl
Total bilirubin	1.14	0.3-1.2 mg/dl
Alanine transaminase	17	<50 IU/L
Aspartate transaminase	21	<50 IU/L
Alkaline phosphatase	82	80-300 IU/L
High-performance liquid chromatography (HPLC)		
Hemoglobin adult (Hb A)	28.4	95-98%
Hemoglobin A2 (Hb A2)	2.0	2-8%
Hemoglobin fetal (Hb F)	11.8	0.8-2.0%
Hemoglobin sickle (Hb S)	57.8	Absent
Immunological tests		
Anti-nuclear antibody	8.85	<25 units
Anti-double-stranded DNA antibody (Anti-DS DNA)	0.35	<0.90
C-reactive protein (CRP) (quantitative)	16.98	<5 mg/ L
Rheumatoid arthritis (RA) factor	Negative	Negative
Antibodies to human immunodeficiency virus (HIV)	Negative	Negative
Anti-cyclic citrullinated peptide antibody (Anti-CCP)	3.05 U/ml	<25 U/ml
Cardiac biomarkers		
Troponin-T (ng/dl)	0.01	0-0.01 ng/dl

**Figure 2:**
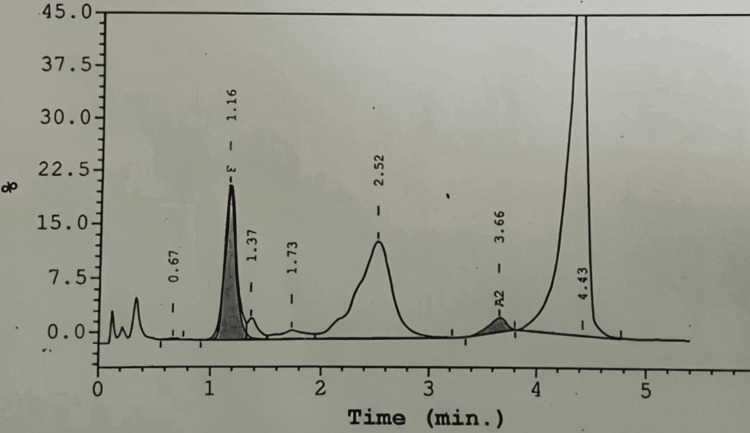
High-performance liquid chromatography (HPLC) showing sickle cell trait

The patient's HPLC findings were as follows: Hb A: 28.4%, Hb A2: 2.0%, Hb F: 11.8%, and Hb S: 57.8%, suggestive of sickle cell trait (Figure 2).

**Figure 3:**
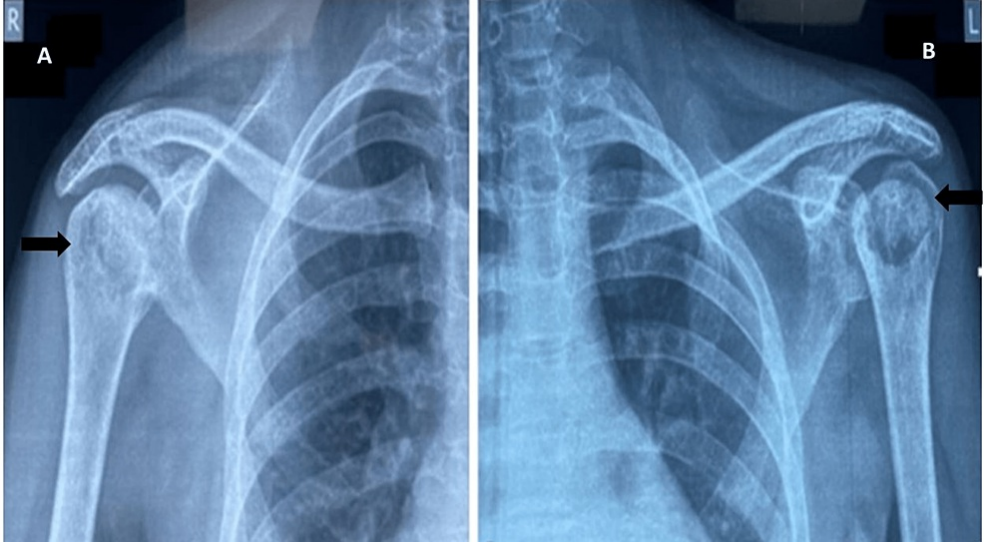
X-ray of right (A) and left (B) shoulders showing osteosclerosis of bilateral shoulder joints (arrows)

X-rays of the left and right shoulder were performed, which revealed bilateral osteonecrosis of the shoulders (Figure 3).

**Figure 4:**
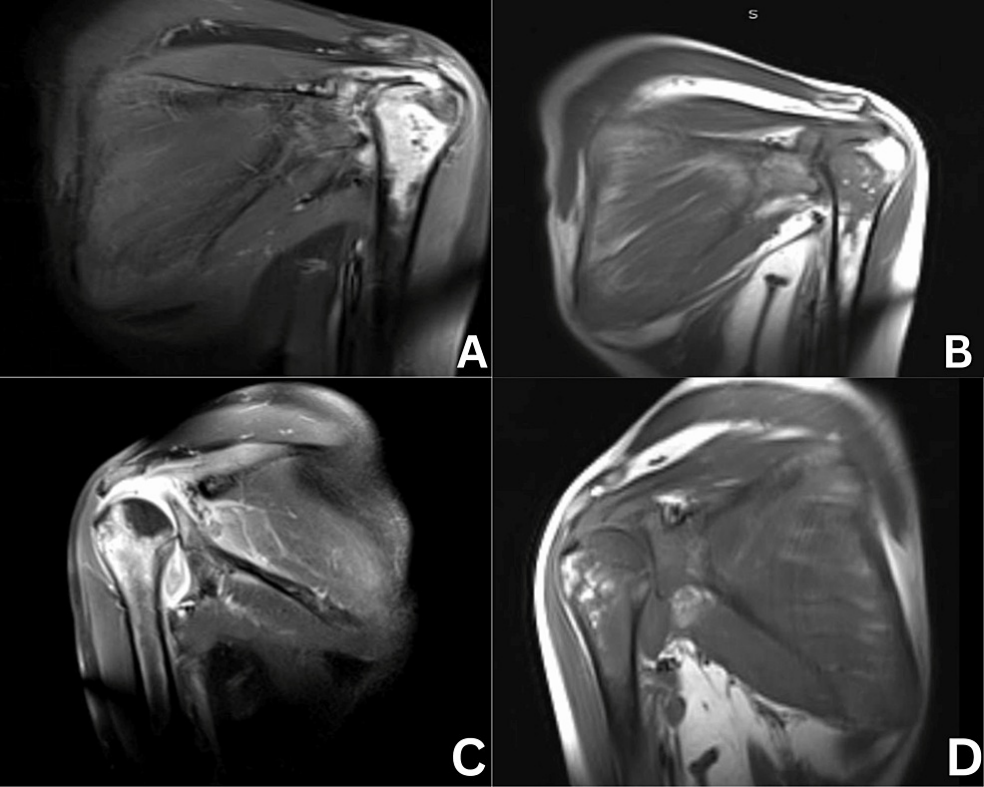
MRI of the patient T1W coronal images showing A: (right shoulder joint) hypointense area involving the cortex and medullary cavity of the head and proximal shaft of right humerus; B: post-contrast enhancement with minimal fluid intensity collection noted in the subacromial subdeltoid bursa and axillary recess; C: (left shoulder joint) hypointense area involving the cortex and medullary cavity of the head and proximal shaft of left humerus; D: post-contrast enhancement with minimal fluid intensity collection noted in the left glenohumeral joint MRI: magnetic resonance imaging

Ultrasonography of the abdomen revealed an empty splenic fossa, suggesting autosplenectomy. The patient also underwent multiplanar, multiecho MRI of both shoulders with and without contrast, which revealed bone infarction with changes related to AVN in bilateral shoulder joints (Figure 4).

The patient was started on intravenous 0.9% normal saline, intravenous morphine 4 mg twice a day for three days followed by tablet ketorolac for pain relief, tablet hydroxyurea 500 mg once a day, and tablet folic acid. An orthopedic consultation was done, and nonoperative intervention along with physiotherapy was advised. Due to the patient's unwillingness to undergo surgical intervention, we continued her on conservative management. She started with physiotherapy. Following the resolution of her symptoms (improvement in pain in both shoulder joints along with an improved range of movement), she was discharged. Even though she was asked to come for a follow-up after one month on an outpatient basis, she was lost to follow-up and subsequent imaging could not be done.

## Discussion

Heimann and Freiberger published the first description of AVN of the shoulder in 1960 [6]. This condition is also called aseptic necrosis or osteonecrosis. AVN may result from a variety of traumatic and non-traumatic causes. Regarding non-traumatic aetiologies, hemoglobinopathies and chronic steroid treatment are common causes, while systemic diseases, including lupus erythematosus, RA, and Cushing's syndrome, are less common [7]. SCD is the most common cause of AVN globally [7]. The sites most susceptible to AVN are the femoral head, femoral condyles, humeral head, capitulum, and the region around the scaphoid and talus [2,8].

Some of the risk factors for the development of shoulder osteonecrosis in SCD patients include hip osteonecrosis, the genotypes hemoglobin S/hemoglobin C or hemoglobin S/beta-thalassemia, and the presence of osteonecrosis in other joints. The rate and risk of osteonecrotic lesion progression to the stage of bone necrosis are closely correlated with the hemoglobin S genotype, a greater osteonecrotic area, and the medial or posterior position of the osteonecrotic lesion [9]. In SCD, due to repeated deoxygenation, RBCs deform into a sickled appearance, which blocks microvasculature, and reduce blood supply to the subchondral bones, leading to hypoxia and cell death. It eventually leads to bone collapse, disintegration of joints, and reduced mobility [10].

MRI is the preferred investigation of choice in conjunction with a comprehensive medical history, physical exam, and evaluation of shoulder radiographs when there is a suspicion of AVN. Altered osteoclast activity decreases bone resorption, as shown on MRI, which is suggestive of osteosclerosis. The T2 signal will rise, and the T1 signal will fall, specifically due to fat cell edema or ischemia of the bone marrow [11]. AVN of the head of the humerus is classified into five stages, as depicted in Table 2, based on the information gathered from MRI and shoulder radiography [6,12].

**Table 2:** Classification of avascular necrosis of the humeral head MRI: magnetic resonance imaging

Stages	Findings on X-ray and MRI of the shoulder	Treatment
Stage I	On T1-weighted images of MRI, bone marrow's hyperintense regions are replaced with hypointensity, whereas a hyperintense focus is visible on T2-weighted images. However, the shoulder X-ray will remain normal	Conservative treatment
Stage II	Sclerotic or diffusely mottled osteopenia, reparative process, preserved sphericity of the humeral head	Conservative treatment
Stage III	Subchondral fracturing, the crescent sign (subchondral radiolucent line), and small depressions in the joint surface may also be present	Core decompression/resurfacing and hemiarthroplasty
Stage IV	Flattening (loss of humeral head sphericity) and collapse brought on by the trabecular pattern's disintegration. Bones with osteocartilaginous flaps and intra-articular loose bodies present	Core decompression/resurfacing and hemiarthroplasty
Stage V	Degenerative arthritis (glenoid articular changes due to joint incongruity)	Hemiarthroplasty or a total shoulder arthroplasty

The management of AVN of the shoulder joint is challenging. Osteonecrosis is a long-term complication of recurrent vaso-occlusive crisis in SCD. Prevention of the progression of AVN can be achieved with drugs like hydroxyurea, which causes an increase in fetal hemoglobin (HbF) and can decrease recurrent vaso-occlusive crises [13]. The advancement of osteonecrosis and the progression to collapse of the humerus head remain unaffected among SCD patients who undergo core decompression surgery for early AVN [5]. The difficulty of managing this patient population is reflected in the varied responses to shoulder arthroplasty [14]. Early diagnosis is vital because joint replacement is unlikely to succeed in later stages. Almost every patient needs opioid analgesics to treat the pain associated with their condition prior to surgery. The episodes of recurrent arterial occlusion in SCD may be a possible cause of the persistence of pain even after arthroplasty. The surgical procedure for humeral head AVN in patients with SCD is chosen based on the stage of osteonecrosis. Prior to collapsing, core decompression is preferred. However, according to the information that is currently available, it has not been proven to stop or slow the usual development and progression of the disease. Despite the fact that shoulder arthroplasty typically has good outcomes in its later stages, data on long-term implant survival and issues are scarce [14,15].

Wong et al. have reported four weekly intra-articular hyaluronic acid injections into the glenohumeral joint over the course of two months as a conservative approach in a case of AVN of the humeral head in the setting of SCD. Short-term benefits were observed in the form of improved physical functioning and a decrease in opiate use. However, more research is needed on the long-term benefits of hyaluronic acid injections in the setting of SCD [16]. The combined effects of core decompression and autologous concentrated iliac crest bone marrow injection were recently studied in SCD patients with stage I and stage II symptomatic humeral head necrosis. These individuals were followed up for 2-10 years, and it was found that their radiological alterations persisted despite an improved range of movement at the shoulder joint [17].

There is currently scarce data on the outcomes of shoulder arthroplasty in sickle cell patients [2,5,7,10,13]. Due to this lack of data, no definitive conclusions can be drawn regarding the effect of prosthesis, hemiarthroplasty versus total replacement, or the use of cement. Although the results were quite diverse and generally less favorable than those of arthroplasty for other types of atraumatic AVN, most patients had an improvement in range of motion after undergoing shoulder arthroplasty. The constantly challenging task of pain control during the perioperative phase is likely what prevents better individual outcomes [18-20].

## Conclusions

We discussed a case of a 25-year-old patient with a recently diagnosed sickle cell trait. Bilateral shoulder AVN at such a young age is a highly uncommon occurrence in sickle cell trait. We evaluated her based on the risk-benefit ratio and current data available on the benefits and complications of total shoulder replacement. We planned to continue to treat her with conservative management and physiotherapy as she had quite a good range of motion preserved in her limbs and given her financial status and unwillingness to undergo invasive interventions. Further research is required to gain deeper insights into the potential link between genetic variations and the success of shoulder arthroplasty.
